# How Ocular Surface Disease Impacts the Glaucoma Treatment Outcome

**DOI:** 10.1155/2013/696328

**Published:** 2013-10-09

**Authors:** Snježana Kaštelan, Martina Tomić, Kata Metež Soldo, Jasminka Salopek-Rabatić

**Affiliations:** ^1^Department of Ophthalmology, Clinical Hospital Dubrava, Avenue Gojka Šuška 6, 10 000 Zagreb, Croatia; ^2^Department of Ophthalmology, University Clinic Vuk Vrhovac, Clinical Hospital Merkur, Zajčeva Ulica 19, 10 000 Zagreb, Croatia; ^3^Department of Ophthalmology, Clinical Hospital “Sveti Duh,” Sveti Duh 64, 10 000 Zagreb, Croatia

## Abstract

The treatment goals for glaucoma are lowering the intraocular pressure and preservation of vision. Topical hypotensive drops are the standard form of therapy which is often associated with some symptoms of toxicity, ocular inflammation, allergy, or ocular surface disease (OSD). OSD is a common comorbidity in glaucoma patients, and its prevalence with glaucoma increases with age. Use of topical treatment could additionally increase symptoms of OSD mostly due to preservatives added to multidose medication bottles used to reduce the risk of microbial contamination. This toxicity has been particularly associated with BAK, the most commonly used preservative which damages conjunctival and corneal epithelial cells and significantly aggravates OSD symptoms. OSD adversely affects patients' quality of life causing discomfort and problems with vision which in turn may result in noncompliance, lack of adherence, and eventually visual impairment. In the management of glaucoma patients OSD symptoms should not be overlooked. If they are present, topical glaucoma treatment should be adapted by decreasing the amount of drops instilled daily, using BAK-free or preservative-free medication and lubricants if necessary. Awareness of the presence and importance of OSD will in turn improve patients' adherence and compliance and thus ultimately the preservation of long-term vision.

## 1. Introduction

Glaucoma is a chronic progressive optic neuropathy usually associated with increased intraocular pressure (IOP). It is characterised by progressive optic nerve damage and functional defects in the visual field which in the final stage can lead to blindness. Recent reports from the World Health Organization indicate that, of the 37 million people who are currently blind, 4.5–5 million people are blind due to glaucoma [1]. Glaucoma is an increasingly common pathology. It is estimated that approximately 60.5 million people worldwide have glaucoma, and it is predicted that the number will escalate to 79.6 million by the year of 2020 mostly due to the rapidly aging population [2]. Its prevalence rate increases with the aging population, namely, in persons older than 40 years, 2.4% have glaucoma, and this further increases to 7% among those older than 70 [3].

Although several risk factors are associated with glaucoma onset and progression, the presence of high levels of IOP is the most important risk factor and the only one that can currently be changed [4]. Therefore lowering IOP is the most efficient and clinically accepted form of therapy used to avoid deterioration of the optic disc and progression of visual loss and thus preserve vision [3]. Despite advances in laser and surgical treatments, topical hypotensive drops remain the standard form of therapy for glaucoma which, as a chronic disease, requires long-term treatment often with multiple ophthalmic medications. There are five categories of topically administered medications available for the treatment of glaucoma, namely, cholinergic agents, adrenergic agonists, carbonic anhydrase inhibitors, *β*-adrenoceptor antagonists, and prostaglandin analogs (PGAs) whose use has shown good efficacy and safety. Whilst topical medication treatment has undeniable advantages and benefits, it also has certain shortcomings particularly concerning its effect on the ocular surface (Table 1) [5, 6].

The tear film is fundamental in the maintenance of the ocular surface. Any condition that adversely affects the stability and function of the tear film such as dry eye symptom, blepharitis, and meibomian gland dysfunction, dysfunctional tear film syndrome, or toxicity of topical medications may result in onset of an ocular surface disease (OSD). The symptoms of OSD may include dryness, burning or stinging, itching, irritation, tearing, photophobia, foreign-body sensation, grittiness, redness, fatigue, fluctuating visual acuity, and blurred vision (Table 2) [9, 10]. Regardless of its underlying case, OSD leads to characteristic pathologic changes in corneal epithelial cells, a decrease in the density of goblet cells, and the upregulation of inflammatory mediators. The visual disturbances and discomfort associated with OSD can be debilitating and often severe adversely affecting the quality of life and patient's physical, social, and psychological functioning [11]. Several factors are considered to influence the prevalence of OSD such as age, sex (particularly female), race, contact lens wearing, vitamin or hormone intake or deficiencies, some autoimmune diseases, and systemic medications such as antihypertensives, antidepressants, and antihistamines as well as topical medications including IOP-lowering agents [10, 12–14].

## 2. OSD in Glaucoma Patients

OSD is a common comorbidity in glaucoma patients in part due to the fact that its prevalence as in glaucoma increases with age. OSD is seen in approximately 15% of the general elderly population and is reported in 48% to 59% of patients with medically treated glaucoma [14–16]. One in six patients with glaucoma has OSD symptoms severe enough that they need some form of treatment [16]. Nevertheless, the diagnosis of OSD in the glaucoma patient is often overlooked since the focus of management is on the evaluation of IOP and markers of glaucomatous disease progression.

Even short-term use of topical glaucoma medications in healthy persons may have deleterious effect on the ocular surface such as reducing corneal sensitivity, tear film stability, or basal secretion [17]. Furthermore in patients with preexisting OSD prolonged topical glaucoma medication treatment additionally deteriorates symptoms [18]. Apart from the known, pronounced severity of glaucoma, glaucoma itself, increasing value of IOP, number of used topical medications, eye drops instilled daily, and history of previous treatment of OSD or history of glaucoma therapy changes due to ocular surface intolerance are also confirmed predisposing factors for OSD in glaucoma patients [12, 13, 18, 19]. Baudouin et al. reported that moderate or severe OSD was found in 63% of patients with severe glaucoma and 41% in those with mild glaucoma with severity directly related to elevated IOP [19]. Furthermore, a considerable difference was also detected based on the type of glaucoma. The eyes with ocular hypertension tended to use fewer eye drops and to have less incidence of OSD. In pseudoexfoliation glaucoma, the prevalence and severity of OSD as well as the number of eye drops were increased compared with those in primary open angle glaucoma (OAG). Eyes with primary angle-closure glaucoma (ACG) had more frequent and severe OSD although the number of eye drops and total dose frequency per day were similar to OAG. However, the reasons for these differences are not yet fully understood. In eyes with neovascular glaucoma OSD was markedly more common and severe where almost all cases had combined proliferative diabetic retinopathy as well as diabetic keratopathy. Therefore, the corneal epithelium in those patients was easily damaged yet more difficult to be remedied [20]. Likewise the glaucoma itself also appeared to be accompanied by a disorder of the tear film and appearance of ocular surface damage. It has been shown that even untreated patients with POAG have reduced basal tear secretion compared with healthy control patients [21].

## 3. Influence of Topical Medication Therapy on Ocular Surface

Topical medication is a very specific treatment in ophthalmological clinical practice since eye drops could have a number of influences that can potentially harm the ocular surface. The volume of one drop is excessive for the conjunctival sac; thus tear components including electrolytes, proteins, and mucin are squeezed out from the tear film which may in turn alter ocular pH and osmotic pressure. Eye drops are composed of the active drug as well as preservatives and a buffer solution, and therefore drug-induced OSD results from the combined influence of the active drug and the vehicle, including preservatives and additives. Certainly any substance instilled into the eye, whether it is an active agent, preservative, or inactive ingredient, has the potential for inducing at least some cellular toxicity and ocular surface changes [20, 22].

### 3.1. Benzalkonium Chloride and OSD

The effect of preservatives in IOP-lowering agents on the ocular surface and their involvement in OSD development have been greatly investigated. Multidose topical ophthalmic medications must include preservative systems in order to prevent microbial contamination and biodegradation and thus maintain drug potency as well as prolong its shelf life [15, 23]. The most commonly used ophthalmic preservative is benzalkonium chloride (BAK) which is the component of approximately 70% of preserved ophthalmic solutions. It is an extremely capable preservative in regard to its microbial coverage and its capacity to break cell-to-cell junctions on the corneal epithelium, thus allowing entry into the anterior chamber and improving the effect of the active drug. BAK is a quaternary ammonium compound that acts as a detergent whose antimicrobial activity arises from its ability to lyse and to disrupt cell membranes. As a detergent, it also impacts the tear film disrupting the lipid layer thereby causing evaporation of its aqueous component. Along with its antimicrobial activity, BAK is toxic to the corneal and conjunctival epithelium as well as the stroma [23]. These toxic activities and changes are both dose and time dependent resulting from its quantity and cumulative impact. BAK remains in the eye long after its application as it incorporates itself into cell membranes for up to 7 days and has a half-life of 12 hours. Standard concentrations range from 0.015% to 0.02% BAK in ophthalmic solutions; however, toxicity has been demonstrated at concentrations as low as 0.005% [24]. Antiglaucoma drops preserved with BAK are also strongly associated with topical inflammation. The microscopic changes seen in patients using BAK include an increase of proinflammatory cytokines, lymphocyte infiltration, fibroblast proliferation, corneal microvilli loss as well as decrease in goblet cell density, epithelial keratinization, and squamous metaplasia which consequently all lead to tear film instability and tear hyperosmolarity [22–26].

### 3.2. Consequences of Chronic Inflammation on Glaucoma Surgical Outcomes

One of the most concerning impacts of the subclinical inflammation caused by glaucoma medications is the failure of filtration surgery, which is frequently a last alternative in the treatment of glaucoma. Several studies maintain that filtration surgery is affected by the duration and the number of glaucoma medications used by the patient [23, 27, 28]. In addition, in patients who have had successful surgeries, much lower preoperative hypercellularity of chronic inflammatory cells (including fibroblasts, macrophages, and lymphocytes) of the trabecular meshwork was found. Therefore there has been solid ground to assume that the presence of chronic inflammatory response associated with preservative toxicity, specifically BAK toxicity, may cause adverse surgery outcome due to fibrosis of the bleb which in turn indicates a strong positive correlation between glaucoma medication and surgery failure [27, 28].

### 3.3. Options for BAK Alternatives

Since it is well known that chronic exposure to BAK-preserved topical IOP-lowering medications is associated with adverse effects on the ocular surface [23, 25], recently glaucomatous eye drops that contain non-BAK preservatives and drugs without preservatives have become available. Several reports have confirmed the advantages of BAK-free antiglaucoma eye drops and demonstrated that adverse reactions induced by BAK-preserved medications may be reversible when administered treatment is changed to BAK-free medications which offer clinically relevant long-term benefits for glaucoma patients [20, 25, 29–31].

There are several other alternative preservatives available designed to eliminate the toxic side effects of BAK in multidose preparations. Stabilized oxychloro complex (SOC) (Purite, Bio-Cide International Inc., OK, USA) as an oxidant preservative also disrupts cellular membranes yet with a milder effect than BAK. The selective bactericidal and fungicidal activities of oxidant preservatives are derived from their inherent ability to create oxidative stress, which eukaryotic cells are able to withstand due to their antioxidant and enzymatic neutralizing ability. Oxidizing agents may lead to less cell damage than detergent preservatives such as BAK. A newer preservative, sofZia (Alcon Laboratories, Inc., Fort Worth, TX, USA) is an ionic, buffered formula composed of zinc, borate, propylene glycol, and sorbitol and has confirmed effective microbicidal activity [22, 23, 25]. Polyquaternium-1 (PQ-1), another potential alternative preservative for use in topical antiglaucoma medication, has been successfully used in contact lens solutions and artificial tears. PQ-1 as a quaternary ammonium compound is a detergent-type preservative derived from BAK but has been shown to be less toxic [25, 32]. Currently, preservative-free topical glaucoma medications which come in single-dose units (SDUs) are also available, and their use allows total avoidance of preservatives with all their associated adverse effects. However, SDU manufacturing and packaging make this type of medications expensive and difficult to use for some patients [33].

Although the majority of glaucoma medications still contain some levels of BAK, recently there is a tendency and recommendation toward alternative preservatives or preservative-free formulations in medical glaucoma treatment. Their use offers clinically relevant long-term benefits to glaucoma patients and may help maintain ocular surface health for an extended period of time [31–33].

### 3.4. Fixed Drug Combination in Glaucoma Treatment

According to the data of Ocular Hypertension Treatment Study, 5 years after the diagnosis of glaucoma, 40% of patients used 2 and 9% of patients used 3 or more topical medications [34]. The likelihood of adverse events increases with multidrug therapy and multiple installations per day, particularly when considering that those containing preservatives can accumulate in the ocular tissue, and thus unfortunately compliance is clearly lacking in many patients. Fixed combinations can be an alternative to maintain adequate IOP control and improve compliance since single-drop therapy is simple and flexible for patients, minimising preservative use as well as the absence of the washout effect present with multiple-drop therapies. Moreover, maintenance of short-and long-term adherence to glaucoma medication regimens is improved with a once-daily fix combination rather than with multiple-drop therapy [35, 36].

## 4. Management of OSD in Patients with Glaucoma

It is an established fact that OSD may be more frequently observed in patients undergoing antiglaucomatous medication treatment. During treatment it is necessary to consider not only the effect of medication on IOP but also the incidence and severity of drug-induced OSD as the most frequent side effect. Careful observation is particularly needed for the eyes that are treated with multiple eye drops and in the older age group [20]. Moderate or severe OSD affected 38% of patients who received a single topical therapy, 54% of those who received 2 topical therapies, and 71% of those who received 3 or more topical therapies [37].

It should however be emphasized that in daily practice the situation is probably even more difficult than that which can be assessed using data available from clinical trials. A proportion of glaucomatous patients with symptoms and signs of OSD is notably higher in clinical practice than in prospective clinical trials or in the general population [10, 19]. The typical duration of clinical trials is 6–12 months, and in this period long-term effects of treatment could be undetected and underestimated. In clinical trials research subjects usually receive only one topical medication whilst in everyday practice medications are often combined. Clinical trials set a washout period (usually one month) before starting or changing eye drop therapy. Conversely, in clinical practice many glaucomatous patients have to continue using one or more eye drops for extended periods of time [20]. Furthermore, clinical trials usually exclude patients who have established dry eye, meibomian gland dysfunction, atopy, and previous ocular allergy or have hypersensitivity to the drug or preservative. Therefore the results obtained from clinical trials indicate better tolerability to topical medication than those found in clinical practice where patients with the mentioned conditions are common [37]. Thus, the background of drug-induced OSD is usually much more severe and difficult to improve in clinical practice than clinical trials actually demonstrate.

These observations have important implications for the medical management of glaucoma patients since coexistence of OSD and low tolerability could act as barriers to compliance with IOP-lowering therapies [15, 16, 38]. Consideration of both efficacy and tolerability should always be prominent in the glaucoma patients' treatment. A routine assessment of glaucoma in clinical daily practice should include an evaluation of OSD symptoms and signs. A comprehensive ocular examination of the ocular surface should be performed on each glaucoma patient before starting a topical therapy as well as during the followup. A few questions and routine check would easily identify the most common symptoms such as feeling of dryness or burning, a foreign body sensation, eyelid hyperaemia, meibomian gland dysfunction, reduced tear break-up time, corneal or conjunctival fluorescein staining, and the need for use of tear substitute [37, 39]. The suggested tests that verify the presence of ocular surface alteration are simple, easy to perform and are not too time consuming (Table 3).

## 5. OSD and Glaucoma Treatment Outcome

We should be aware that the symptoms of OSD can severely impair glaucoma patients' functioning and quality of life with discomfort being an important aspect to consider in the followup [9, 11, 16, 40, 41]. Topically treated glaucoma patients with OSD recorded lower quality-of-life scores versus patients without surface damage. Patients who have less expressed symptoms of OSD may be more likely to adhere to recommended treatment, thereby effectively lower their IOP, and decrease glaucomatous vision loss, respectively [39, 40]. Previous episodes of moderate-to-severe OSD symptoms frequently require a change of antiglaucoma treatment. Switching to a different topical medication or systemic therapy with carbonic anhydrase inhibitor, or performing laser trabeculoplasty or other surgery along with adding treatment to alleviate ocular surface symptoms is possible. Modification of glaucoma therapy due to OSD is applied in approximately 40% of patients [37]. The reasons for discontinuation or lack of adherence with glaucoma treatments may be associated with the type of medication, patient beliefs, disease characteristics, and ultimately satisfaction with treatment [42, 43]. Certainly one of most common reasons for discontinuation or inadequate adherence to glaucoma treatment is the development of OSD [15, 23].

Despite the side effects, their impact on visual disturbances, and discomfort, both ophthalmologists and glaucomatous patients have to tolerate mild symptoms in order to continue topical medications due to characteristics of glaucoma treatment and the severity of glaucoma consequences. Mild OSD without any related subjective symptoms may be permissible and tolerable during glaucomatous treatment, and the use of lubricants in the form of tears, gel, and ointment particularly BAK-free lubricants to alleviate those symptoms could be useful [20].

Poor compliance was found in more than one-third of patients with glaucoma depending on the type and duration of therapy which was further pronounced with advancing age [4, 16, 38]. It is an established fact that adequate glaucoma therapy requires a high level of compliance, and this can certainly be better achieved with a lower use of ocular medications [44]. The ocular surface status should be evaluated regularly before starting a new chronic topical therapy and during the followup to ensure the timely detection and treatment of pathological signs on the ocular surface. It should be kept in mind that BAK may act as an anaesthetic and may mask the experience of subjective symptoms associated with OSD [45]. Misdiagnosed and, consequently, untreated OSD may adversely affect patients, quality of life since it causes significant discomfort and more importantly problems with vision which is undoubtedly affected by a change in the quality of the tear film itself. Adding to this the exacerbating effects of topical glaucoma medications on ocular surface aggravate the patient's treatment compliance and therefore consequently reduce adherence [37, 46]. Motivation to adhere to prescribed therapy may already be low since early glaucoma itself is a silent disease, and compliance is likely to be low when perception of treatment is less tolerable than the disease itself. Furthermore in the long term, poor treatment response due to discomfort caused by topical medical glaucoma treatment may lead to irreparable visual loss.

In conclusion, several variables including patients' familiarity and understanding of glaucoma, beliefs in medication efficacy, cost of treatment, and awareness of medication side effects as well as the impact of noncompliance play a vital role in glaucoma treatment outcome. The availability of a broad spectrum of options for glaucoma treatment will subsequently enable more personalized and effective patient care. Selecting a therapy regimen that does not conflict with patients' well-being or decrease their motivation for treatment nor their quality of life will significantly enhance glaucoma treatment and thereby facilitate preservation of long-term vision. Moreover, awareness of the importance of OSD prevalence and its adverse effects and consequences in glaucoma patients should undeniably be reflected on the selection of the available treatment options.

## Figures and Tables

**Table 1:** Topical glaucoma medications: systemic and ocular side effects.

	Systemic side effects	Ocular side effects
Nonselective beta-blockers	Decreased heart rate, bradycardia, arrhythmias, exacerbation of heart failure, masking of hypoglycemic symptoms, and depression	Burning, redness, decreased ocular blood flow, and decreased corneal sensation

Alpha-2 agonists	Hypotension, respiratory depression (in infants), central nervous system depression (in infants), sedation, and fatigue	Redness, itching, pupillary dilatation, and lid retraction

Carbonic anhydrase inhibitors	Allergy, bitter taste, and low blood counts	Stinging, irritation, and red eyes

Prostaglandin analogs	No significant side effects	Hyperemia, changes in particularly skin pigmentation, changes in iris color, and eye-lash growth

Table information adapted from [7, 8].

**Table 2:** Symptoms and signs of ocular surface disease.

Symptoms
Visual disturbances
Dry eyes sensation
Tearing
Burning
Foreign-body sensation
Stinging or burning sensation
Eyelid itching
Photophobia
Grittiness
Frequent/repeated blinking

Signs

Abnormal Schirmer test
Abnormal tear osmolarity
Abnormal tear break-up time test (TBUT)
Meibomian gland dysfunction
Turbid meibomian gland secretions
Lid margin vascularization
Lid margin laxity and/or irregularity
Corneal and/or conjunctival staining
Limbal and/or bulbar hyperaemia

**Table 3:** Evaluation of the ocular surface.

Subjective assessment	
Questionnaires:	
OSDI: ocular surface disease index	
GQL-15: glaucoma quality of life-15	

Clinical testing	

Visual acuity	
Assessment of morphology of the eyelid and meibomian gland	
Assessment of meibomian gland secretion	
Tear break-up time test (TBUT)	
Corneal and conjunctival staining (fluorescein/lissamine green/rose bengal)	
Schirmer test	
Tear film osmolarity	
